# Considering young and socially marginalized people in urban revitalization: evidence on enablers and barriers from a mid-size Canadian city

**DOI:** 10.3389/ijph.2026.1609821

**Published:** 2026-09-28

**Authors:** Martine Shareck, Clara Blache-Pichette, Jeremy Hugonnier, Meghan Winters, Anne-Marie Tougas, Daniel Fuller

**Affiliations:** 1 Département des sciences de la santé communautaire, Université de Sherbrooke, Sherbrooke, QC, Canada; 2 Faculty of Health Sciences, Simon Fraser University, Burnaby, BC, Canada; 3 Département de psychoéducation, Université de Sherbrooke, Sherbrooke, QC, Canada; 4 Department of Community Health and Epidemiology, University of Saskatchewan, Saskatoon, SK, Canada

**Keywords:** local governance, structural determinants of health, urban renewal, urban revitalization, youth

## Abstract

**Objective:**

This study explored what enabled and constrained consideration of young and socially marginalized people’s needs and interests in the downtown revitalization of a mid-size Canadian city.

**Methods:**

In this interpretive descriptive study, we conducted nine semi-structured interviews with 10 key informants from the municipal, community organization and private sectors involved in the revitalization initiative. We analyzed interview transcripts following the framework method to identify main themes.

**Results:**

According to participants, young and socially marginalized people’s needs and interests were not explicitly considered since the initiative predominantly aimed to improve the downtown physical and economic environment. Participants were, however, open to collaborate for a more equitable and inclusive revitalization, but diverging perspectives on (1) reasons for revitalizing the downtown, (2) how “young” and “socially marginalized” people were implicitly defined, (3) collaborative structures and mechanisms, and (4) public participation processes and strategies, constrained fulsome consideration of these groups.

**Conclusion:**

The factors we identified as enabling and constraining consideration of young and socially marginalized people who experience neighbourhood revitalization firsthand can serve as a springboard to devise solutions for inclusive and health-promoting urban planning.

## Introduction

Many cities worldwide are deploying important neighborhood revitalization initiatives. These involve a combination of small- and large-scale built environment interventions including the creation of green and public spaces, road upgrades, transportation improvements, and building of new housing, retail, and workspaces [1]. Such interventions are necessary to improve aging infrastructure and build resilience against climate change [2, 3]. Since it targets important social determinants of health, neighbourhood revitalization can also support health and wellbeing by improving access to essential services, promoting safety and mobility, and fostering opportunities for social interaction and physical activity [4–8]. It may, however, also have negative impacts including social network disruption [9, 10], exclusion and displacement of socially marginalized groups [11], and gentrification [12, 13]. Notably, certain social groups such as younger and older adults, women, ethnic minorities, and people who are unemployed or live on a low income may not benefit from neighbourhood revitalization as much as their counterparts, which may consequently lead to social inequalities in health [10, 14, 15].

Several aspects of revitalization initiatives influence whether they may reduce or increase social inequalities in health. This includes their underlying objectives, the types of interventions they consist of, where they are implemented, and who participates in decision-making and how. These aspects are shaped by local governance, a key structural determinant of health defined as the set of collective rules and processes, formalized or not, through which key actors take part in decision-making and implementation of public policy and action [16, 17].

In line with a structural determinants of health perspective, several dimensions of local governance shape the inclusive and equitable character of urban revitalization initiatives, i.e., one that ensures a fair distribution and access to health-promoting resources for all social groups, and in which everybody can fully participate [18] and, ultimately, who benefits from them and who does not [18, 19]. For example, given that revitalization initiatives are usually developed outside the health sector and overseen by municipal authorities, but that they involve additional actors from a diversity of sectors, collaborative and intersectoral governance models can help integrate health, social, economic, and environmental goals [20] and reconcile sectoral interests and expectations. Models which engage communities and citizens–particularly residents of areas undergoing revitalization, and especially those who belong to socially marginalized groups–through adequate public participation mechanisms also support revitalization initiatives that better address local needs [15, 20, 21] and are more likely to support health.

On the other hand, several factors may constrain inclusive and health-promoting revitalization, including difficulties enacting intersectoral collaboration due to unequal power relations [21, 22] or conflicting frames of reference and logics in how actors understand revitalization objectives, target populations, and impacts [23]. Difficulties meaningfully engaging socially marginalized groups, including young people, in participatory processes [21, 22, 24, 25], sometimes because of systemic marginalization of these groups from decision-making in urban planning [26, 27], represent another oft-cited challenge to inclusive and health-promoting revitalization.

Most studies investigating local governance in the context of neighbourhood revitalization have focused on large cities. This has limitations since small- and mid-size cities are distinct from larger ones in terms of human and financial resource availability, power dynamics, and proximity to local populations, which may lead to different local governance issues [28]. Evidence generated from larger cities may not transfer directly to revitalization processes in smaller urban contexts [29], even though these play a central role in countrywide revitalization. In Canada alone, approximately 25% of the population lives in small- and mid-size cities (i.e., <500,000 inhabitants) [29], many of which are investing significant resources to revitalize their downtown areas [30, 31]. Documenting local governance challenges in ongoing revitalization initiatives of small- and mid-size cities is essential for guiding the development and implementation of health-promoting and inclusive revitalization initiatives [2, 32].

The current study was part of a large mixed-methods project evaluating a multi-phase revitalization initiative of the downtown area of a mid-size Canadian city. Here we specifically explored what enabled or constrained the consideration of young and socially marginalized people’s needs and interests in the first phase of the initiative. We focused on these groups for several reasons. First, they were over-represented among downtown residents and users, as described below. Second, young and socially marginalized people already face multiple challenges related to housing, employment, and income–three social determinants of health plausibly affected by revitalization efforts. As such, they are more likely to be negatively impacted by revitalization efforts than other socio-demographic groups. Third, these groups tend to be under-represented in municipal engagement and decision-making, so research is needed to better understand how to include them in such processes [33, 34].

## Methods

### Context: downtown revitalization initiative

In 2020, a mid-size city of approximately 200,000 inhabitants in the province of Quebec, Canada, began revitalizing its downtown area [35]. In 2016, the downtown area, which covers 1.1 km^2^, was home to approximately 3,020 residents, 34.1% of which were young people between 18 and 29 years old. Census data further highlighted the precarious conditions in which downtown residents lived. All age groups combined, 93.3% were renters and 57.1% lived below the poverty line. Furthermore, downtown residents had a median after-tax household income three times lower (CAD 16,617$/year for 15 year-olds and older) and unemployment rates twice as high (51.4% of 25–64 year-olds) compared to the city as a whole. [36]. These statistics guided the focus of the present study on young people, namely young adults apprixmately 18 to 30 years-old, and on individuals who are socially marginalized due to living in precarious housing, employment and income situations.

In the first phase of the revitalization initiative, 80 million Canadian dollars were invested (25 million dollars from public funds) to build two new towers housing 60 residential rental units, office and retail space, a rooftop garden and a daycare center, develop a public space atop an indoor parking lot, and improve streetscape aesthetics [35]. These changes were hypothesized to positively impact downtown residents and users through increased housing availability and proximity to services, as well as opportunities for social connections in the newly built public space. According to a public consultation report, planned changes were generally welcomed by local community organizations and residents who were nonetheless concerned regarding potential negative impacts for young and socially marginalized individuals who were over-represented in the neighbourhood [37]. Whether planned changes would improve the social conditions and health of long-term residents was deemed to depend on whose needs and interests were considered, and how, in the planning and implementation of the initiative [37], which is what we explored here.

### Study design and data collection

We conducted an interpretive descriptive study embedded within a larger mixed-methods evaluation project. In Summer 2022, a research assistant performed nine semi-structured interviews with 10 key informants. Participants were identified by the larger project’s advisory committee composed of representatives from academia, municipal staff and community organizations, through a document and news article review, and snowball sampling. We selected participants for their knowledge of the revitalization initiative and/or work with young and socially marginalized people. Interviewees represented municipal staff (n = 3) and elected officials (n = 2), community organizations (n = 3), private builders (n = 1) and advocacy groups (n = 1). The interview guide (Supplementary Material 1) covered four broad topics: (1) participant’s role in the revitalization initiative and its underlying purpose, (2) intersectoral collaboration (e.g., “What has been done to promote collaboration between the different actors... involved in this project,…?) (3) inclusion and consideration of young and socially marginalized people in the initiative (e.g., What factors contributed to, or hampered, inclusion of [young people] or [socially marginalized groups] and consideration of their needs?), and (4) challenges and impacts surrounding the development and implementation of the initiative. Topic 3 was introduced by providing summary statistics for the downtown population related to young and socially marginalized people as described above. This was done to provide participants with a working definition of these groups. In-person or video interviews (25–60 min) were conducted in French, digitally recorded, transcribed verbatim and checked for accuracy. Interviewees provided informed consent prior to data collection. This study received ethical approval from CIUSSS de l’Estrie-CHUS

### Analysis

We analyzed interview transcripts following the framework method [38], a structured approach recommended for multidisciplinary teams like ours, with experts in public health, social work and political science. The analysis consisted in (i) transcription; (ii) data familiarization; (iii) coding; (iv) developing a working analytical framework; (v) applying the analytical framework deductively; (vi) charting the data into the framework matrix; and (vii) interpreting the data. To develop the analytical framework, two research assistants CBP and JH independently and inductively coded a sub-sample of transcripts, keeping the objectives and interview guide in mind. They revised each other’s coding and discussed any questions and discrepancies with the first author MS to reach consensus. The final analytical framework was then applied deductively to all transcripts.

We relied on several strategies to ensure study quality as per Tracy et al. (2019) Credibility was enhanced by recruiting participants from diverse sectors, allowing for multivocality, i.e., the inclusion and representation of multiple, potentially disagreeing, voices. Rich rigor and credibility were further supported by iterative, team-based coding following a structured approach as described above. Sincerity was supported through research logbooks and systematic documentation of questions, feelings, interpretative working notes, and decisions made [39].

## Results

Our study explored what enabled or constrained the consideration of young and socially marginalized people’s needs and interests in the first phase of a large downtown revitalization initiative. We found participants were open to collaborating towards an inclusive approach to downtown revitalization which would integrate the needs and interests of young and socially marginalized people, over-represented downtown. However, we documented diverging perspectives on four themes which seemed to constrain their fulsome consideration: (1) tensions surrounding reasons for revitalizing the downtown area; (2) diverging definitions of “young” and “socially marginalized” people; (3) contrasting experiences of collaborative structures and mechanisms; and (4) inappropriate public participation processes and strategies. These diverging perspectives merit being reconciled if a more inclusive and health-promoting revitalization is to be pursued. We describe and discuss each theme below.

### Tensions surrounding reasons for revitalizing the downtown area

First, participants suggested that young and socially marginalized people’s needs and interests were not explicitly considered because the revitalization initiative was driven by a desire to improve the downtown physical infrastructure and local economy, rather than by concerns for social equity. Participants agreed the downtown area was in disrepair and understood that developing its economy was essential for attracting new residents, businesses, and workers who would stimulate its vitality, while positively impacting the surrounding neighbourhoods and city. However, pursuing infrastructural and economic interests, at the detriment of social equity, was seen as problematic by some interviewees:

« Socially, the problem with this downtown revitalization is that it's not a real downtown revitalization, it's more like an investment to advance certain economic interests. »

(Participant 5 – Municipal - Elected official).

It also led to criticisms and concerns from both the municipal and community organization sectors regarding unintended consequences on local residents, a large proportion of which was already living in precarious conditions. According to these participants, additional efforts should have been made to explicitly address the needs of young and socially marginalized people since one could not expect revitalization benefits to trickle down to these more vulnerable groups:

“[…] given the amount invested in this project, I cannot believe we did not think about investing in securing the people who were going to be victims of the consequences of this project […] taking care of poverty is not that expensive…”

(Participant 2 – Community organization)

Consequently, several participants felt that key actors were evolving in “parallel worlds”, as one representative from the community organization sector put it: one in which the downtown’s state of disrepair and decreasing economic vitality justified, in itself, the revitalization, and another in which the needs and interests of young and socially marginalized groups who lived and spent time downtown should more fully be considered so as to allow them to remain in the neighbourhood.

### Diverging definitions of “young” and “socially marginalized” people

Participants also differed in how they spoke about and implicitly defined “young” and “socially marginalized” people. In some cases, these subgroups overlapped, but not always. In general, “young people”, as a population, encompasses a wide array of individuals, and accordingly, participants each tended to refer to a specific type of young person based implicitly on their personal experiences. Regarding “socially marginalized” people, participants defined them as individuals living in poverty or those who were unhoused. Differing views arose, however, regarding who was responsible for looking after young and socially marginalized groups and the potential solutions to support them.

For example, several participants suggested the new infrastructure would serve most young people who lived and spent time downtown, even though they were not explicitly targeted by the project. Some mentioned the planned rooftop garden and daycare center were especially tailored to young people, defined here as young professionals and families living downtown. These same participants also argued that the new rental housing and employment opportunities in the retail and video game industry were developed with the whole city’s population in mind, including younger age groups. On the other hand, community organization representatives suggested this population-wide approach failed to address the needs of a specific subgroup of young people over-represented downtown, namely those living on a low income, in precarious housing or who struggled with mental health conditions. This subgroup of socially marginalized young people risked being excluded from the new infrastructure tailored to young people in general, and forced to leave the neighbourhood to fulfill their daily living needs:

“A 5 1/2 room apartment in an office tower, with a view of the river, with home automation at $2,000 per month, I don't know anyone I work with who needs that. Was it created for these people [socially marginalized young people]? I don't think so. It was created to bring people downtown; I think the end goal was to transform the downtown landscape by bringing in people who are not here, but very little has been done for the people who were already here.” (Participant 2 – Community organization)

This discrepancy in defining key populations potentially affected by the revitalization was compounded by the fact that the rooftop garden and daycare center did not materialize in the early phases of revitalization. The implementation process therefore contributed to unmet expectations regarding catering to young people, regardless of whether they were defined as young professionals and families or as more socially marginalized individuals.

### Contrasting experiences of collaborative structures and mechanisms

Contrasting experiences of collaborative structures and mechanisms established as part of the revitalization initiative also limited the consideration of young and socially marginalized people. Early on, the city created an intersectoral committee to favour dialogue and consensus-seeking amongst the diversity of actors who operated downtown or were concerned by its revitalization: “… entrepreneurs, merchants, community groups, cultural groups, citizens, and residents who lived directly in the downtown area, and … people from health institutions … All these people … were around the table.” (Participant 4 – Municipal–Elected official).

This collaborative governance structure was viewed as enabling the integration of diverse actors’ opinions and interests in the revitalization initiative. Municipal and private actors considered it particularly legitimate given the privileged information that was discussed in meetings and the influence participants could have on decision making. On the other hand, community organization representatives reported that final decisions seemed to be taken by the city and private developers (who were partially funding the project). For this reason, they felt they ultimately had little influence on decisions being made:

“Very concretely, in the day-to-day [sic], we have no role to play in the development of this project. We've been invited to sit on committees and things like that. […] but I don't think we can boast of having any impact on any institutional decision-making afterwards.”

(Participant 2 – Community organization)

As a result, community organizations’ unique expertise regarding young and socially marginalized people was not fully harnessed. This limited the full and early consideration of the diversity of young and socially marginalized people present in the downtown area. It may also have led to proposing “band-aid” solutions to address their needs, such as installing public restrooms for unhoused individuals or putting young people in charge of keeping a new public space clean to engage them in the project. Ultimately, these solutions minimally contributed to the revitalization initiative’s inclusiveness. Although the governance structure that was privileged showed potential for supporting intersectoral collaboration and inclusion, it fell short due to contrasting experiences regarding whose expertise was valued and who was involved in decision-making.

### Inappropriate public participation processes and strategies

A fourth theme concerned public participation processes and strategies. Participants described multiple strategies used to inform and gather the public’s opinion on the revitalization initiative. Information and progress regarding the initiative was communicated to the public through social media, local radio campaigns and the City website where project orientations and timelines were shared. City staff also consulted the public through events such as a co-creation design charette as early as 2017. Subsequent activities such as the 2020 “question-and-answer” online event were, however, less participatory and focused on sharing information.

Despite this diversity of public participation strategies which, in theory, could enable reaching a wide array of people, participants voiced concerns regarding the methods prioritized. They highlighted that strategies were not tailored to young and socially marginalized people who lived downtown. For example, they mentioned that promotional campaign organizers seemed to assume that social media would naturally reach these groups, although many did not have access to the Internet nor to a social media account. The municipal sector seemed aware of social media shortcomings, as expressed by one participant:

“We used social media a lot more because we know today that, increasingly, a lot is won through social media […] but you have to be careful because we’re excluding part of the population who does not have access to Internet. You always need to have a little flyer lying around in display stands […] You need a little bit of everything.”

(Participant 4, Municipal – Elected official).

Despite this awareness, many participants recognized that those in charge of public participation activities failed to rely on alternate means of communication. For example, community organization representatives noted that basic outreach, such as advertisements for public participation activities posted near the construction site which was directly opposite housing for socially marginalized young people, was lacking:

“For me, working here and downstairs where the young people live, it was never clear, for example, posted outside: “Next information session…” and all that. If you didn't go looking for the information and if it didn't come to you by chance, it wasn't very accessible…»

(Participant 8 – Community organization)

Participants also mentioned that common barriers to public participation, such as social precariousness, over-solicitation, and mental health issues were not considered when designing activities, despite their high prevalence among downtown residents. These barriers further constrained young and socially marginalized people’s ability to shape the revitalization initiative. They also compounded the fact that important decisions seem to be made regardless of what the public shared, with little transparency and accountability towards public input:

“You go to a public event, people throw out ideas, you discuss them, and then, after that, not much is retained, or only some of the ideas are retained. If young people had wanted to be involved, it would have had to be in a more structured way.”

(Participant 5 – Municipal - Elected official)

In sum, the collaborative governance structure established tended to overlook the critical insights of young and socially marginalized groups’ representatives, and the public participation strategies privileged fell short to include them specifically. Nevertheless, all participants recognized that the revitalization initiative’s lack of social inclusiveness likely stemmed from shortcomings related to knowledge of young and socially marginalized people’s reality, rather than from conscious attempts to exclude them. As one participant put it, key actors’ openness to dialogue might enable deeper intersectoral collaboration in the future:

“Still, there was a desire to understand. There's a lot of education to be done on both sides, I think; there's a lack of understanding of the issues of poverty; you cannot blame anyone if they've never rubbed shoulders with these people. It's interesting to know that there's an open door on the city side to discuss these issues.”

(Participant 2 – Community organization)

## Discussion

Anchored in a structural determinants of health perspective [18, 19], this paper explored what enabled and constrained consideration of young and socially marginalized people’s needs and interests in the downtown revitalization of a mid-size Canadian city. We identified diverging perspectives between actors involved in the downtown revitalization regarding four key topics related to local governance, and found these to constrain the fulsome consideration of young and socially marginalized people’s needs and interests in the initiative.

First, we found that the revitalization initiative was driven by political and private actors motivated by a desire to improve the area’s physical and economic environment rather than by social equity concerns. This is not entirely surprising given that revitalization initiatives are often driven by urban planning and private interests rather than by health and equity concerns [9, 14]. It also aligns with studies of other types of urban transformations, such as greening interventions–which are not usually centered on socially marginalized residents *per se* [14, 40] – but also from studies of community-led initiatives which, by design, often focus on social equity and inclusion [25]. This is nevertheless unfortunate since neighbourhood revitalization directly impacts the social determinants of health and, ultimately, may lead to social inequalities in health [10]. As such, social and health-related objectives should be explicitly integrated at the outset of these initiatives to support more inclusive and equity-focused transformations [20, 40].

Second, we found that discrepancies in how participants implicitly defined “young” and “socially marginalized” people and what they perceived their needs to be, undermined collective efforts to reach those who could benefit the most from the intervention such as young downtown residents who lived in precarious conditions. Similar to other urban revitalization initiatives [25, 41, 42], this lack of consensual definitions of key populations may have prevented anchoring dialogue and decisions in a shared understanding of issues and potential solutions [25, 43], and acted as a barrier to a more socially inclusive initiative. Anchoring urban interventions in the social context of place is essential for equity-focused action [15, 43]. Moving forward, defining key terms, establishing a clear understanding of local residents’ characteristics and needs, and centering all phases of revitalization initiatives, including implementation, around social equity and inclusion would be useful [24, 25].

Third, in our study, an intersectoral committee was established to facilitate collaboration between municipal, private, and community actors. Such structures require members to step outside their comfort zone and sectoral silo to engage in dialogue and debate, and achieve a common understanding of key issues and solutions [44]. Here, this proved difficult at times, with evidence of diverging goals and definitions as discussed above. Additional challenges included unequal power dynamics leading to asymmetrical influencing and decision-making power, and inadequate accountability towards committee members and the general population. These commonly arise in urban transformation initiatives [45, 46], with the most powerful actors, such as those from the municipal and private sectors, often shaping narratives and taking final decisions [24, 46], as perceived here. Previous studies have also discussed other pitfalls we documented regarding the lack of accountability concerning the uptake (or disregard) of suggestions that would promote social inclusion within revitalization initiatives. In the future, these challenges could be addressed through trust building [44], broader participation of all interest holders including local communities [47], and transparency in decision-making through all phases of the revitalization initiative [47]. These strategies could support more inclusive collaboration and decision-making that integrate the concerns and aspirations of all parties involved.

Finally, public participation activities were seen as falling short of including young and socially marginalized people, in part because they were rooted in assumptions about these groups’ literacy level (assumed to be high) and preferred modes of communication (assumed to be social media). Activities were also perceived as taking place *after* major decisions had already been made, leaving little room for influencing them. Although commonplace in urban revitalization endeavours [20, 21, 40, 48], this contrasts with public participation ideals which call for early and sustained engagement [49], especially that of those most likely to experience intervention impacts firsthand [20, 40]. We also found that public consultation–where information was shared unidirectionally–were more common than co-construction participatory strategies. While not uncommon, this approach can lead to revitalization initiatives that overlook structural problems and reinforce social and health inequalities [24, 48, 50]. Inclusive participatory processes is notably challenging and can be constrained by strict laws and regulations [50], the time and effort required to organize events and develop accessible contents [25, 51], power differentials [24], and difficulties reaching marginalized groups and sustaining their engagement [24, 25, 52, 53]. Nevertheless, solutions exist. In our study, young and socially marginalized people’s engagement could have been supported by relying on accessible and diverse communication strategies [15, 43], including outreach [25, 43, 53], by training citizens, municipal and private actors in inclusive communication and collaboration [25, 43], and by being accountable for including these groups [26].

### Strengths, limitations and future research

Study strengths include our focus on a mid-size city, often overlooked in urban planning and health research, and on an ongoing intervention, which limited participants’ recall bias. This timing also meant that findings could guide the planning of subsequent phases of the downtown revitalization initiative. Interviewing representatives from a diversity of sectors ensured we covered a wide range of viewpoints. However, we would ideally have interviewed young and socially marginalized individuals themselves, in addition to community organizations representing them. However, our sampling approach did not identify any key individuals who could have participated on their own behalf. The present findings must therefore be interpreted as illustrative of community organization representatives’ perspectives, which may differ from that of individuals *per se*. As part of another aim of the overarching research project, we conducted individual interviews with downtown residents which provide complementary insights into young and socially marginalized people’s lived experiences of the revitalization and associated public participation processes. This will help address the above limitation. Finally, it was beyond the scope of this study to examine the direct link between local governance and downtown residents’ health, but this would be worth exploring in future research, such as with longitudinal studies.

### Conclusion

Urban revitalization requires integrating a plurality of actors and their assumptions, experiences, and interests. This complexity exposes the limits of traditional governance models and their difficulty in reconciling diverging perspectives to the benefit of socially marginalized populations who, oftentimes, are most directly impacted by urban transformations. Different aims and visions of urban revitalization lead to different action priorities and, consequently, unequal social and health benefits. Our findings underscore the need for early and sustained commitment to addressing young and socially marginalized people’s needs, and for innovative collaborative governance and public participation structures and processes, for urban transformations to be health-promoting and socially inclusive.
