# Supplementary material for: Considering young and socially marginalized people in urban revitalization: evidence on enablers and barriers from a mid-size Canadian city
Source: Int J Public Health. 2026 Sep 28;71:1609821. doi: 10.3389/ijph.2026.1609821 (PMC13654851; doi:10.3389/ijph.2026.1609821)
Supplement: Supplementary file 1 [file DataSheet1.pdf]

Key Informant Interview Procedure and Guide

Urban environment and health among young adults in Sherbrooke

Purpose of the interview

Individual interviews will focus on the development and implementation of the downtown Sherbrooke revitalization initiative, the key informant's role in the initiative, and their perspective on the factors that contributed to or hindered consideration of young and socially marginalized people's needs and interests in the development and implementation of the initiative. Conducted in-person or virtually, interviews are expected to last 30 to 60 minutes and will be digitally recorded. Interviewees will provide verbal or written informed consent.

Interview procedure

Consent, Preamble, Explanations and Answering questions

Read the key informant interview information and consent form. Allow interviewees to ask questions and then get their verbal consent.

Share this image on the screen so that the interviewee has a visual representation of the revitalization initiative of interest.

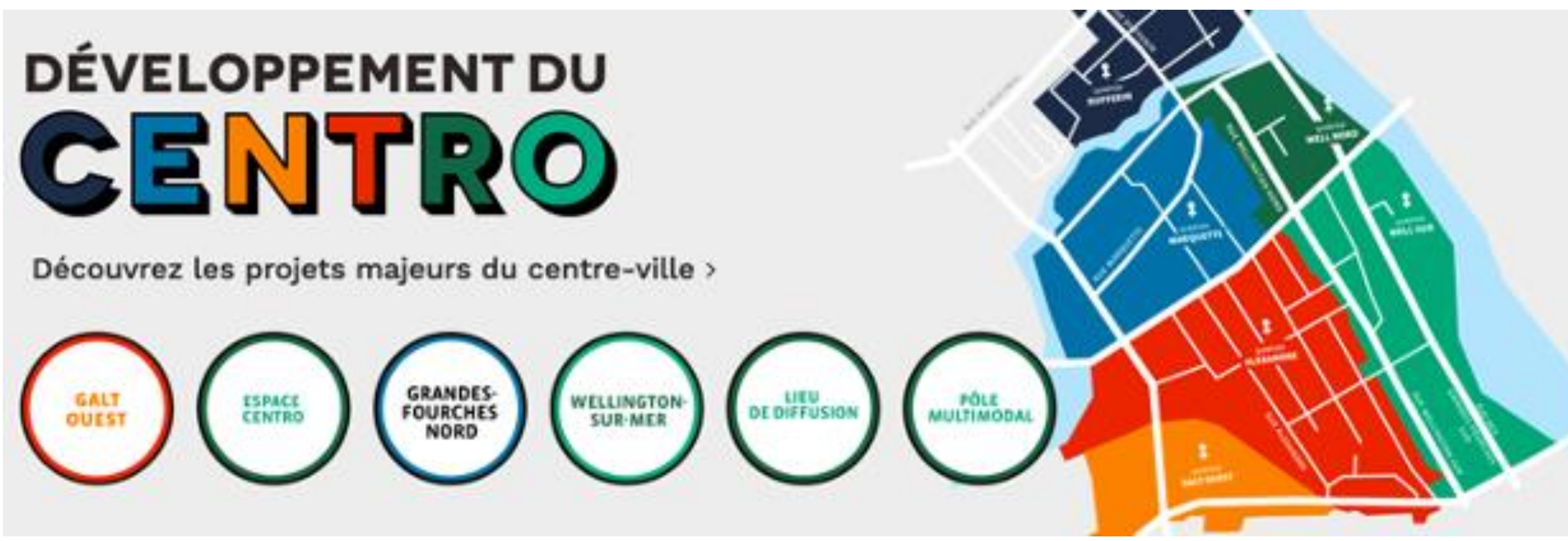

"This is a map of major projects that are underway or planned for downtown Sherbrooke. We are interested in changes in the physical environment of the downtown core as a whole, but during the interview, we will discuss more specifically the "Espace Centro" intervention program in the Well Sud neighbourhood.

During the interview, we will discuss the following themes: your role in the project, collaboration between the different actors involved in the revitalization initiative, inclusion of young and socially marginalized people (e.g., young adults, lower-income people, those who are homeless, etc.) in the development and implementation of the project, and past and anticipated challenges and obstacles in the initiative's implementation.

If you need a break at any time, don't hesitate to tell me. If there are any questions you don't want to answer, you don't need to answer them. That's totally fine. There is no right or wrong answer. Do you

have any questions before we start? I will now record the interview to facilitate the transcription afterwards."

***** Start Recording *****

Topic 1. Role of the key informant in the initiative

With the first topic of the interview, we would like to better understand the role you have played and/or are currently playing in the revitalization initiative.

1. First, could you briefly introduce yourself (name, organization, role)?

2. What has been your involvement in the downtown Sherbrooke revitalization initiative, and more specifically in the "Espace Centro" project in the Well Sud neighbourhood over the past few months or years?

Follow-up questions:

- What role have you played/are you playing?
- How long have you been involved?
- What was/is the nature of your involvement (committees, meetings, activities, etc.)?
- What made you get involved?

3. In your opinion, what is the overall value of this project for downtown Sherbrooke?
   - Revitalization in general? The Espace Centro project in particular?
   - … for the city?
   - … for your organization/partners?
   - … for neighbourhood/community residents?

4. Have you heard of any groups that are dissatisfied with this project?
   - (For example, a Facebook group "Against the gentrification of Sherbrooke" has recently been created...)
   - What did you hear?

5. Are you communicating with others about this project?
   - If so, what are the target audiences and the nature of the communications?

Topic 2 Collaboration between the different actors

Several groups of stakeholders are concerned by the development of the downtown core and have been or are involved in one way or another in the planning stages of the project (for example, intersectoral revitalization committee, public consultations, etc.).

6. In your opinion, what has been done to promote collaboration between the differnt actors (municipal, commercial, community organiaztions, citizens) involved in this project (e.g. communications, consultations, consultations, decision-making, etc.)?

– What's going well?
– What could be improved or done differently?
– What is the group dynamic?

7. What are the collaborative challenges the project faced (planned and unforeseen)?
   – What worked well? Not well?
   – What could be improved?

Topic 3. Inclusion of young and socially marginalized people in the initiative

Young adults between the ages of 18 and 29 make up 34.1% of the downtown population. Data from the 2016 census highlights the precarious conditions of downtown residents: 93.3% are renters, 57.1% live below the poverty line (compared to 17.7% in the city), the median household income is three times lower than for the city as a whole, and the unemployment rate is twice as high as for the city as a whole. In addition, 16% of residents report having poor mental health, compared to 10% in the Eastern Townships, and primary care interventions for mental health are 7 times higher than elsewhere in the region.

8. In your opinion, have these typical characteristics of the downtown area been taken into account in the development, and more recently in the implementation, of the revitalization initiative?
   – If so, how?
   – If not, what could be the consequences, in your opinion?

9. To your knowledge, knowing that the downtown attracts a lot of young people (adolescents and young adults) as residents and visitors (through schools, shops, services that cater to them), has this age group taken a large enough place in the development of the initiative?
   – If so, how? What has been done to include them and consider their needs and interests?
   – If not, what could be the consequences for young people? What could have been done? What could be done in the future?
   – In your opinion, would young people have wanted to be (more) involved?

10. What are the factors that have contributed to or hindered the inclusion of young people and the consideration of their needs?
    – Contributed
    – Impeded

11. Some groups are more marginalized than others because of low income, unemployment, or homelessness. What are the factors that have contributed to or hindered the inclusion of socially marginalized groups and the consideration of their needs?

Topic 4. Challenges and impacts surrounding the development and implementation of the initiative

The last topic of the interview aims to properly document the contextual elements to better understand the evolution of the initiative and the next steps.

12. What is exciting for you about the evolution of this initiative?

13. What are your concerns about the evolution of this initiative?

14. What issues would be important to pay particular attention to in the coming months/years?
    - What do you expect from the coming months/years?

Interview conclusion

15. What do you think is the revitalization initiative's biggest success to date?

16. The interview is now done. Is there anything else you would like to share with me that we haven't covered yet?

***** Stop recording *****

[Examples of clarifying questions include:

- Can you tell me a little more about it?
- Can you tell me more about it?
- Can you give me some examples?
- How do you explain...?]
